# Nanoporous Ni with High Surface Area for Potential Hydrogen Storage Application

**DOI:** 10.3390/nano8060394

**Published:** 2018-06-01

**Authors:** Xiaocao Zhou, Haibo Zhao, Zhibing Fu, Jing Qu, Minglong Zhong, Xi Yang, Yong Yi, Chaoyang Wang

**Affiliations:** 1 School of Materials Science and Engineering, Southwest University of Science and Technology, Mianyang 621010, China; zhouxc529@gmail.com (X.Z.); qm5739@163.com (J.Q.); 2 Research Center of Laser Fusion, China Academy of Engineering Physics, Mianyang 621900, China; fuzhibingcn@163.com (Z.F.); 13110200008@fudan.edu.cn (M.Z.); xingxingysx@163.com (X.Y.); 3 Center for Degradable and Flame-Retardant Polymeric Materials, College of Chemistry, National Engineering Laboratory of Eco-Friendly Polymeric Materials, Chengdu 610064, China; haibor7@163.com

**Keywords:** nanoporous Ni, high surface area, hydrogen storage

## Abstract

Nanoporous metals with considerable specific surface areas and hierarchical pore structures exhibit promising applications in the field of hydrogen storage, electrocatalysis, and fuel cells. In this manuscript, a facile method is demonstrated for fabricating nanoporous Ni with a high surface area by using SiO_2_ aerogel as a template, i.e., electroless plating of Ni into an SiO_2_ aerogel template followed by removal of the template at moderate conditions. The effects of the prepared conditions, including the electroless plating time, temperature of the structure, and the magnetism of nanoporous Ni are investigated in detail. The resultant optimum nanoporous Ni with a special 3D flower-like structure exhibited a high specific surface area of about 120.5 m^2^/g. The special nanoporous Ni exhibited a promising prospect in the field of hydrogen storage, with a hydrogen capacity of 0.45 wt % on 4.5 MPa at room temperature.

## 1. Introduction

Presently, as a kind of novel structural and functional material combining metallic materials’ properties (such as electric and thermal conductivity, ductility, and catalytic activity) and porous materials’ properties (such as low density, high porosity, high specific surface area, and high strength-to-weight ratio) [1,2], nanoporous metal materials have aroused intense interest for a wide range of important potential applications. These include battery-like supercapacitors [3], high power density batteries [4], viable hydrogen storage [5,6], electromagnetic composites [7], filtration and desalination [1], fuel cells [8,9], sensors [10], microwave adsorption [11], and magnetic media [12]. Nanoporous metals are more difficult to prepare in comparison with other nonmetallic nanoporous materials, owing to the difficulty in generating wet metal gels [13,14]. Hence, only a few synthetic pathways have been demonstrated to prepare nanoporous metals. So far, approaches for the preparation of nanoporous metals include combustion synthesis, self-assembly, dealloying, and template synthesis [2]. Notably among them, combustion synthesis requires unconventional conditions such as high temperature and pressure and oxygen-/water-free environments [1]. Self-assembly is often used to fabricate nanoporous noble metals but nanoporous nonprecious metals (Ni, Co, Cu), which have wide applications in science and industry, are difficult to synthesize in this way [8,15,16]. In contrast, dealloying has been widely applied to prepare nanoporous metals but it demands expensive sacrificial metals [13,17]. Nevertheless, these methods lead to limitations in acquiring hierarchical pores within nanoporous metals, which are important to the application of hydrogen storage and fuel cells [1,18]. 

Templating is known as a powerful method to control porosity and porous structures within metals by using sacrificial inorganic or organic materials as templates [2], so it is the most commonly used method for preparing nanoporous metals with desirable structures [19,20]. Hsueh et al. [21] deposited Ni into a block copolymer via electroless plating to prepare nanoporous gyroid nickel with a surface area of 25 m^2^/g. Jiang et al. [22] demonstrated an electroless deposition technique in penetrating the interstitial spaces of template (colloidal crystal) that was extendable to a variety of materials including Ni, Cu, Au, Pt, and Ag. Based on a similar technique, Huang et al. [23] attempted to prepare nanoporous Ni by using degradable melamine-formaldehyde (MF) aerogels as a template and the surface area of the nanoporous Ni was 53.8 m^2^/g. Although these templates are often used for the preparation of nanoporous metals, it is difficult to obtain large-area continuous nanoporous metallic bulks or films. Furthermore, the purity of the resultant nanoporous metals is low due to the incomplete removal of the template. Moreover, nanoporous metals prepared with these templates also suffer problems resulting from the limitations of low surface area (<100 m^2^/g) and single pore structure. In fact, the surface area and hierarchical pore structures of nanoporous metals are of importance for practical applications. Recently, SiO_2_ aerogel with a three-dimensional continuous nanoporous structure not only has a porosity of up to 99.0% but also has a high specific surface area range of 200–1000 m^2^/g. It can also be dissolved and removed with NaOH/HF at moderate conditions [24], which can be used as an ideal template for preparing nanoporous metals with considerable surface area and desirable pore structure.

Nickel, in particular with nanostructured textures, has been widely used in science and industry, especially in the field of catalysts, magnetic media and sensors [21]. Besides, with the properties of highly selective adsorption of hydrogen, large specific surface area, high surface reactivity, high catalytic efficiency, and strong adsorption capacity, nanoporous Ni can be used for hydrogen storage [25,26]. However, few works on the preparation and properties of nanoporous Ni with surface area in excess of 100 m^2^/g and hierarchical pore structure has been reported to date. So far, there are some approaches to prepare Ni (nanoparticles, nanowires, etc.)/SiO_2_ aerogels including the incipient wet impregnation and precipitation methods [27,28], both of which focus on the fabrication of Ni/SiO_2_ aerogel composites with a small amount of Ni dispersed in the SiO_2_ aerogel. Compared with these methods, the electroless plating method can make a large amount of Ni particles continuously grow in the pores and channels of SiO_2_ aerogel, finally leading to the formation of nanoporous Ni with a three-dimensional network structure after removing the SiO_2_ aerogel.

In this manuscript, we report a facile method to create nanoporous Ni with superior specific surface area by using soluble SiO_2_ aerogel as the template. The influence of different electroless times and temperatures on the structure, morphology and magnetism of porous Ni were studied in detail. The resultant nanoporous Ni with hierarchical pores and high specific surface area (120.5 m^2^/g) was used for hydrogen storage research and exhibited excellent capacity for hydrogen adsorption.

## 2. Materials and Methods

### 2.1. Materials

In the present work, the preliminary materials were tetraethyl orthosilicate (TEOS, Tianjin Kemiou Chemical Reagent Factory, Tianjin, China). Other chemicals including PdCl_2_, HCl, NiCl_2_·6H_2_O, KOH, hydrazinium hydroxide (N_2_H_4_·H_2_O, 80%), ammonia (NH_3_·H_2_O, 85%), and acetone were supplied by Chengdu Chemical Industries Co. (Chengdu, China). All chemicals used in this experiment were of analytical grade and used as received without any further purification. Twice distilled water was used throughout the experiment.

### 2.2. Preparation of SiO_2_ Aerogel

SiO_2_ aerogels were synthesized through the sol-gel process and CO_2_ supercritical drying. Briefly, TEOS (11.2 mL), EtOH (23.4 mL), and H_2_O (18 mL) were mixed with continuous stirring until the solution became transparent, then a few drops of HCl was added to adjust the pH value of the mixed solution to 3.0. The obtained solution was transferred into a hydrothermal reactor and subsequently placed into a drying oven at 80 °C for about 3 h to obtain the gel. Finally, the SiO_2_ aerogel could be obtained after the gel was exchanged with acetone and dried via supercritical CO_2_.

### 2.3. Preparation of Nanoporous Ni

First, SiO_2_ aerogels were placed in the activating solution with PdCl_2_ (0.05 g), ethanol (45 mL) and HCl (1 M, 5 mL) for 24 h and rinsed with distilled water several times to remove PdCl_2_ enriched on the hydrogel surface. Then the samples were transferred to the nickel plating solution with NiCl_2_·6H_2_O (0.2 g), ethanol (5 mL), distilled water (20 mL), ammonia (2 mL) and hydrazinium hydroxide (2 mL) to start electroless plating at 35 °C until the solution became colorless. The samples were immersed in fresh plating solution for 3/6/9 times to obtain SiO_2_/Ni composites with different theoretical contents of Ni (0.15/0.30/0.45), denoted as SiO_2_/Ni-1, SiO_2_/Ni-2, SiO_2_/Ni-3, respectively. After that, SiO_2_ templates were dissolved and removed by NaOH (5 M) solution at room temperature to obtain nanoporous Ni named Ni-1, Ni-2, and Ni-3, corresponding to SiO_2_/Ni-1, SiO_2_/Ni-2, SiO_2_/Ni-3, respectively.

### 2.4. Characterization

The crystallographic structure of the resulting materials was characterized by X-ray diffractometer (XRD, X’Pert Pro), using Cu Kα radiation. Inductively coupled plasma (ICP) spectroscopy was carried out using Varian 715-ES equipment. X-ray photoelectron spectroscopy (XPS) was carried out on an XSAM 800 spectrometer (Kratos Co., Manchester, UK) with Al Kα excitation radiation (1486.6 eV) and the binding energy was referenced to C 1s at 284.6 eV. The scanning electron microscope (SEM, Nova, 600i), equipped with an energy dispersive spectrometer (EDS) and transmission electron microscope (TEM, JEM-200CM, 200 kV), was used to measure the morphology and structure of the samples. Nitrogen physisorption measurements were carried out at 77.3 K using Quantachrome Autosorb-1 Instruments. The pore-size distributions of nanoporous Ni samples were determined by the Barret-Joyner-Halenda (BJH) method and Density-Functional-Theory (DFT) method and the specific surface area was measured by the multipoint Brunauer-Emmett-Teller (BET) theory. The surface area analyses were performed in the relative pressure of 0.05–0.3. Total pore volume was defined as the volume of liquid nitrogen corresponding to the amount adsorbed at a relative pressure P/P_0_ = 0.99. The magnetic properties were measured with a vibrating sample magnetometer (bkt-4500z). Hydrogen adsorption measurements of the nanoporous Ni were carried out with magnetic suspension balance (Isosorp HP Static I) at room temperature and in the pressure range of 1–5 MPa by using a high-pressure apparatus. The unit was calibrated with high-purity He at various initial pressures. The samples were degassed by vacuum heat treatment (120 °C for 12 h) to remove impurities such as adsorbed water, before being exposed to hydrogen and then the sample was cooled to room temperature. During the experiment, room temperature was maintained at (25 ± 1) °C.

## 3. Results

### 3.1. Structural Characterization of Nanoporous Ni

**Figure 1:**
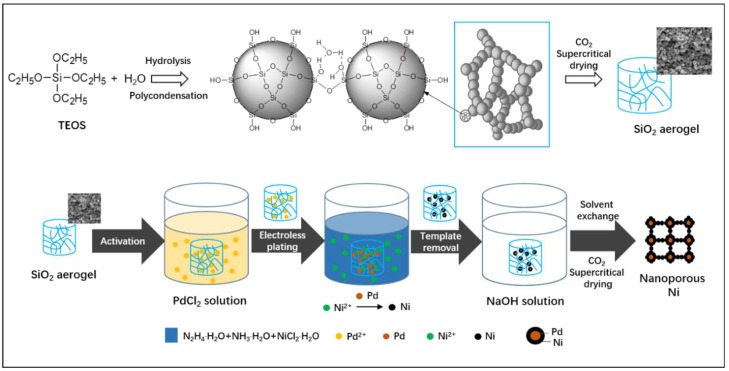
Schematic illustration for fabrication of SiO_2_ aerogel and nanoporous Ni.

Figure 1 illustrates the fabrication route of nanoporous Ni using SiO_2_ aerogel as templates. SiO_2_ aerogel with a 3D porous network and high surface area (890 m^2^/g) was synthesized via a sol-gel process (Figures S1 and S2). Then, a two-step chemistry process was employed to fabricate the Ni aerogel (Figure 1). Ni nanoparticles were chemically deposited on the surface of SiO_2_ aerogel templates by an autocatalytic reduction process [21,29]. During the reduction process, Pd particles were first deposited on the surface of the SiO_2_ aerogel as the catalytic agent. Subsequently, Ni ions were reduced into Ni nanoparticles via N_2_H_4_·H_2_O at the activated area, as shown in Figure 1. After that, the SiO_2_ aerogel templates were dissolved and removed by NaOH solution. Finally, the nanoporous Ni could be obtained after the resultant samples were exchanged with acetone and dried by supercritical CO_2_.

**Figure 2:**
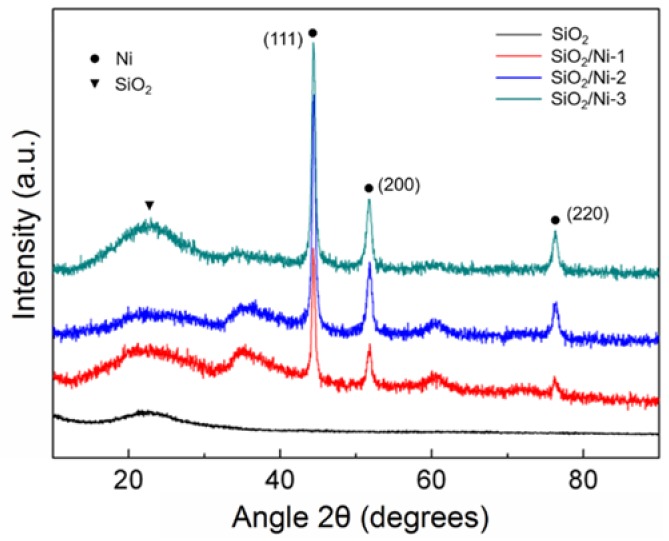
XRD spectrums for SiO_2_/Ni composites.

The crystalline phase of the composites was confirmed by XRD measurements. The XRD patterns corresponded to three kinds of SiO_2_/Ni composites at different times of the electroless plating process and pure SiO_2_ aerogel (Figure 2). A wide-angle peak at 2θ = 23° could be observed from all the samples, which was identified for the typical amorphous SiO_2_ XRD pattern. Except for SiO_2_ aerogel, the other SiO_2_/Ni samples showed three intensity peaks at 44.4°, 51.8°, and 76.3°, which were well indexed to (111), (200), (220) reflections of face-centered cubic crystal structure (JCPDS card NO.04-0805). From this, it can be concluded that Ni particles were successfully incorporated into SiO_2_ aerogels with the existence of crystalline Ni. The crystallite size could be calculated based on the full width at half maximum of the (111) reflections (Scherrer equation) from the XRD spectra. The average crystallite sizes of the obtained Ni particles in the SiO_2_/Ni composites with different electroless plating time were 28.17 nm, 35.7 nm, and 79.5 nm, respectively, indicating a correlation between the increase of the average crystallite size of Ni with the increase of electroless plating time.

The microstructures of the SiO_2_/Ni composites prepared at different electroless plating times at a temperature of 35 °C are depicted in Figure S4. It is clear that the SiO_2_/Ni composites were all highly porous with 3D network structures. With the increase of electroless plating time, the size of particles became larger from 20 nm to approximately 100 nm. There were no obvious bright and dark areas in Figure S4a,b, which means the uniform distribution of Ni nanoparticles in SiO_2_ template at the electroless plating time of 3 times. It can also be observed that an agglomeration exists with the increase in electroless plating time, especially in the plating time of 9 times (Figure S4e–f). In order to further study the pore structure of the SiO_2_/Ni composites, nitrogen adsorption tests on all samples were carried out. The nitrogen adsorption-desorption isotherms and corresponding DFT pore size distribution curves are shown in Figure S5. The N_2_ adsorption curves for composites prepared by electroless plating at different times are similar to that of pure SiO_2_ aerogel and both present type IV isotherms with H3 hysteresis loop, indicating that SiO_2_/Ni composites still maintain the porous structure characteristics of the SiO_2_ aerogels after reaction. It can be seen from Figure S5b that the pore size distribution of the samples before and after electroless plating are basically the same, but as the electroless plating time increased, the amount of the same pore size distribution area gradually declined. In particular, with the increase of time to 9 times, there was no existence of mesoporous in the sample. Table S1 shows the specific surface area (S_BET_), total pore volume (V_t_), and average pore size of the samples calculated using the Brunauer-Emmett-Teller (BET) method, based on nitrogen adsorption-desorption isotherms. It can be concluded from the table that the S_BET_, V_t_, micropore volume, and average pore size of the sample decreased after electroless plating when compared with that of pure SiO_2_ aerogel, indicating that the Ni deposited on the SiO_2_ aerogel and the pores of the aerogel template were partially filled with Ni. Moreover, with the increase of the electroless plating time, the content of Ni deposited on the pores of the SiO_2_ template increased, which resulted in a decrease of the S_BET_, V_t_ and average pore size of the sample.

**Figure 3:**
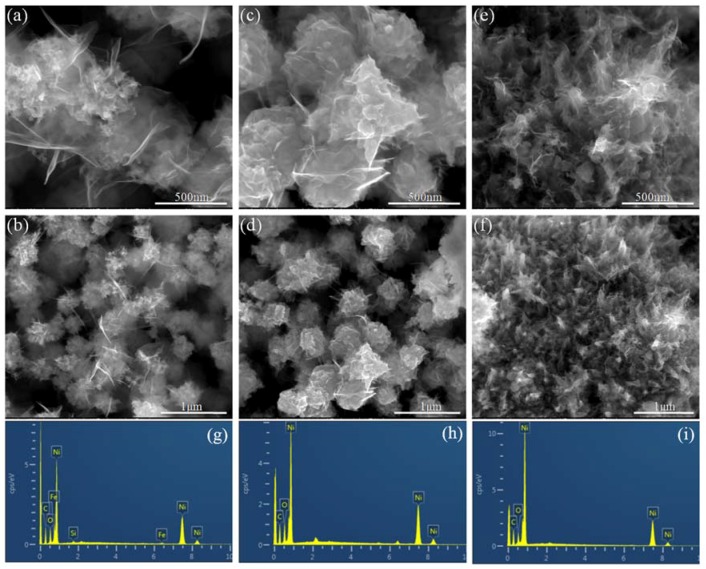
SEM images of the nanoporous Ni obtained with different electroless plating times at 35 °C: (**a**,**b**) 3 times; (**c**,**d**) 6 times and (**e**,**f**) 9 times. EDS spectrums for nanoporous Ni: (**g**) 3 times; (**h**) 6 times and (**i**) 9 times.

The microstructures of the nanoporous Ni derived from the removal of the SiO_2_ template were characterized by SEM. The SEM micrographs (Figure 3) indicated that all of the nanoporous Ni samples were highly porous with a three-dimensional network of irregular interconnected particles. The nanoporous Ni was rich in hierarchical pore with a distribution range from mesoporous to microporous including independent and continuous open porous cells (2–50 nm). Interestingly, it is worth noting the special porous flower-like structure comprised of nanoparticles and the ultrathin sheets around the particles. It can be seen from the SEM images (Figure 3b,d,f) of the nanoporous Ni obtained after removing the template that the size of Ni particles increased with the electroless plating time. There was no accumulation of Ni particles in Figure 3a, but it was obvious that the Ni particles continued to grow and then formed larger particles observed from Figure 3c. While the sample in Figure 3e tended to grow in the closed-cell structure, almost no open-cell structure can be observed from Ni-3. As the reaction continued, the autocatalytic action of Ni caused the reaction to proceed preferentially on the surface, so that the nickel on the surface of the template grew and linked together, thereby agglomerating or even forming a closed-cell structure. Energy dispersive X-ray spectrum (EDS) analysis was performed for all the nanoporous Ni with different plating times, and the typical spectrums are shown in Figure 3g–i. It was clearly observed that the nanoporous Ni was mainly composed of Ni with trace amounts of Si, C, and O. Combined with the results of inductively couple plasma optical emission spectrometry (ICP-OES, Table S2), it is clear that the purity (~95.0%) of the resultant nanoporous Ni is quite high. It can be concluded that the nanoporous Ni is mainly composed of Ni element and the SiO_2_ aerogel template has been removed thoroughly via NaOH solution.

**Figure 4:**
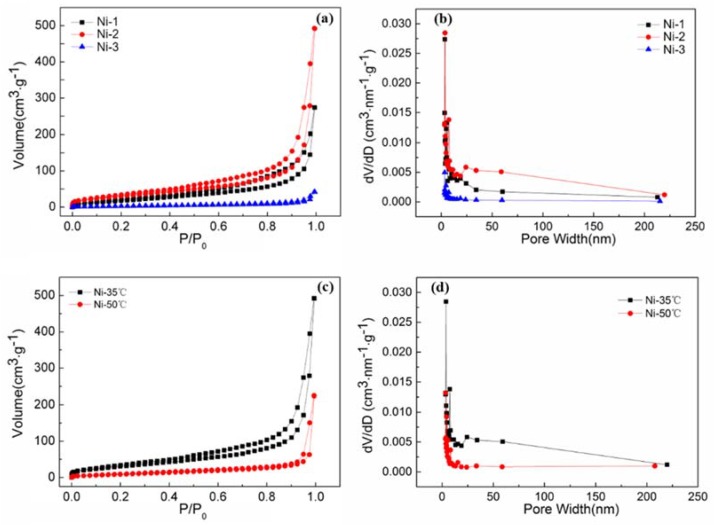
Typical nitrogen adsorption and desorption isotherms and size distribution based on the Barret-Joyner-Halenda (BJH) method for the nanoporous Ni at different electroless plating times (**a**,**b**) and for Ni-2 at different electroless plating temperatures (**c**,**d**).

To investigate the influence of electroless plating time and temperature on pore structure of nanoporous Ni, the nitrogen absorption-desorption isotherms and corresponding pore size distribution curves were investigated, as shown in Figure 4a–d. Figure 4a illustrates the nitrogen adsorption-desorption isotherms of samples obtained by 3, 6, and 9 times of electroless plating at 35 °C. Both samples Ni-1 and Ni-2 had similar isotherm curves but different adsorption-desorption volumes. Additionally, the volume for nitrogen adsorption on Ni-2 was 500 cm^3^/g, which was about 2 times as high as that of Ni-1 at the identical adsorption conditions. Type IV isotherms with H1 hysteresis loops indicated typical mesoporous structures for those two samples. The pore size distribution was further determined by the BJH (Barret-Joyner-Halenda) method. From Figure 4b, it can be seen that most of pores lay in the range below 30 nm, revealing that all the samples mainly contained micropores and mesopores. It should be noted that a few macropores (pore size in the range of 50–100 nm) existed in the Ni-2 sample. However, few macropores at the same size range could be found in the Ni-1 sample, which is consistent with SEM results. Meanwhile, compared with that of Ni-1, Ni-2 showed a stronger intensity of mesopores. This could be attributed to the fact that the nickel particles continued to grow and then formed a three-dimensional porous structure with the increase of plating time, which is beneficial to the formation of mesopores and macroscopes (50–100 nm). It is worth mentioning that there were almost no pores in sample Ni-3, which was due to the agglomeration of Ni particles with the increase of plating time. Analysis of the nitrogen adsorption-desorption isotherms (Figure 4a,c) and the parameters of textural characteristics of nanoporous Ni are shown in Table 1. The BET-specific surface areas of Ni-1, Ni-2 and Ni-3 were 76.79 m^2^/g, 120.54 m^2^/g and 13.48 m^2^/g, respectively. Therefore, it could be clearly seen that electroless plating time had a great influence on the specific surface area of the nanoporous Ni. In particular, high specific surface area exceeding 100 m^2^/g could be found in nanoporous Ni-2, which is higher than the highest values (~75 m^2^/g) ever reported of nanoporous metal materials [15]. 

The effects of electroless plating temperature on pore structure of nanoporous Ni were also studied. Figure 4c,d show the nitrogen adsorption-desorption isotherms and pore distribution curves of nanoporous Ni obtained with electroless plating time of 6 times at temperatures of 35 °C and 50 °C, respectively. As shown in Figure 4c, the amount of nitrogen adsorption of Ni-50 °C was much less than that of Ni-35 °C. Noted that the number of mesoporous was also quite a few and almost no macropores existed in sample Ni-50 °C (Figure 4d). It can be concluded from the Table 1 that the specific surface area and total pore volume decreased with the rising of electroless plating temperature. This can probably be attributed to the fact that the rate reaction of nickel ions increased rapidly and the controlled degree of the reaction decreased with the increase of temperature. The nickel particles deposited in the SiO_2_ framework were more prone to agglomeration at higher temperatures, thereby reducing the number of pores and decreasing the specific surface area of the nanoporous Ni. Hence, the temperature of electroless plating is also a key factor in obtaining nanoporous material with a high surface area.

### 3.2. Magnetic Properties of Nanoporous Ni

**Figure 5:**
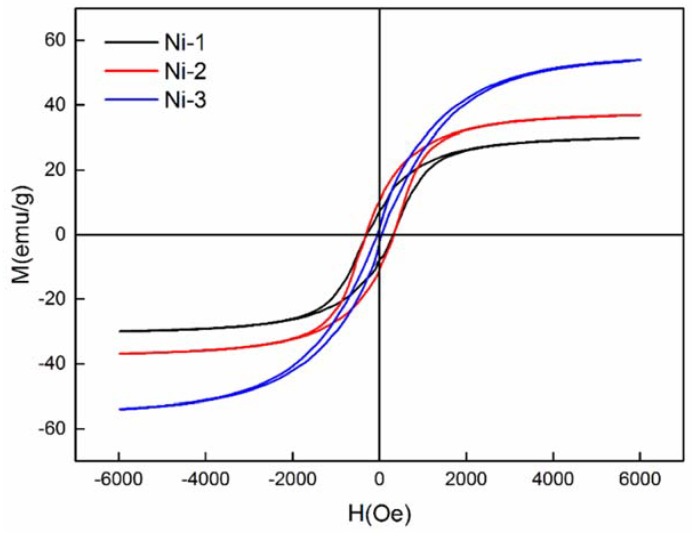
Magnetic hysteresis loop of nanoporous Ni with different plating times at 35 °C.

As we know, nickel is one of the most important magnetic materials. To further analyze the magnetic properties of the resultant nanoporous Ni, magnetic measurements on three samples prepared with different electroless plating times at 35 °C were carried out in the applied magnetic field range (−6000 Oe–6000 Oe) at room temperature. The hysteresis loop of the nanoporous Ni was measured at room temperature as shown in Figure 5. Observation of hysteresis curves indicates that the products obtained exhibited ferromagnetic properties [30]. Table 2 shows the magnetic parameters, including saturation magnetization (Ms), remnant magnetization (Mr), and coercivity (Hc) of the three samples and pristine bulk Ni. We can clearly observe that the Ms value of the samples shows a tendency of decreasing first and then increasing with electroless plating time, which may be caused by the change of the grain growth orientation with time. 

In addition, since the nanoporous Ni was not a single-phase nanoparticle, the nickel crystal grain increased, resulting in the decrease of coercivity, when the electroless plating time increased. Compared with the Ms, Mr and Hc values of the pristine bulk Ni (55 emu/g, 2.7 emu/g, 100 Oe) at room temperature, the Ni-1 and Ni-2 presented a distinctly enhanced coercive force. The higher coercivity in Ni-1 and Ni-2 might have been due to the smaller particle size and the higher anisotropy of the crystallite arrangement [31]. Meanwhile, the value of Ms of the Ni-1 and Ni-2 were less than that of bulk Ni, owing to small crystalline Ni nanostructures [32]. The technological applications of magnetic materials are dominated by their magnetic properties. As for data storage devices, the stable switchable magnetic states of materials are required [12]. The lower saturation magnetization and enhanced coercivity of nanopronous Ni mean that it is favorable for application in data storage devices.

### 3.3. Hydrogen Adsorption

**Figure 6:**
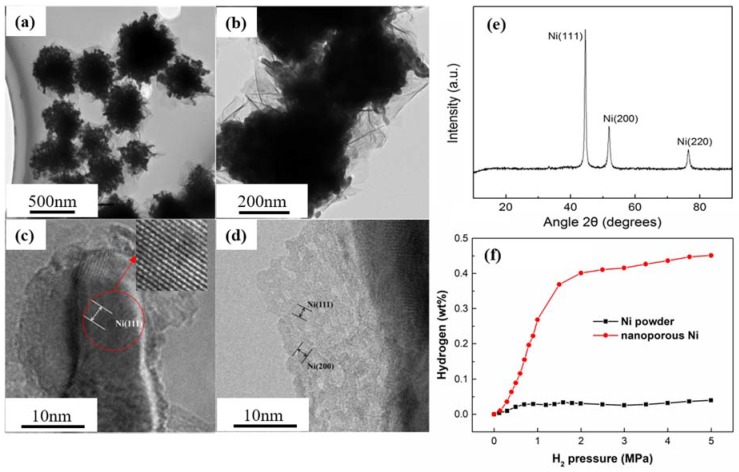
TEM (**a**,**b**), HRTEM (**c**,**d**) images and XRD pattern (**e**) for nanoporous Ni with the plating time of 6 times at 35 °C; (**f**) hydrogen adsorption curves for the nanoporous Ni and Ni powder at room temperature.

In order to further characterize the structure of nanoporous Ni with the highest specific surface area obtained after electroless plating 6 times at 35 °C, a TEM and HRTEM were used, as shown in Figure 6a–d. Then; the hydrogen storage capacities of the nanoporous Ni were measured by magnetic suspension balance. 

From Figure 6a,b, it can be seen that the network-like porous structure of the nanoporous Ni prepared by this method was formed by the deposition of Ni nanoparticles, while the Ni nanoparticles grew centering on the nucleus and formed a thin layer-like morphology. Figure 6c,d show high-resolution transmission electron microscope (HRTEM) images of nanoporous Ni, where (c) was the HRTEM image of Ni particles and (d) was that of multi-layer or single layer sheets. As shown in Figure 6c, we can see that the lattice fringe of the intact Ni particles is clearly visible and the distance between the crystal planes is 0.203 nm through calculation, corresponding to the Ni (111). From Figure 6d, it can be seen that there are obvious lattice fringes in the HRTEM images of the multilayered lamellar morphology but their distribution was more complicated and the growth of crystal plane was disordered. By calculating and comparing with the standard card, the interplanar spacings were 0.202 nm and 0.179 nm, which correspond to the (111) and (200) crystal planes of face-centered cubic (fcc) nickel, respectively. That is to say, the lamellar materials are also a morphology of nickel. The X-ray diffraction (XRD) pattern revealed the crystal structure of nanoporous Ni obtained with electroless plating 6 times. The results are shown in Figure 6e. It was found that the amorphous peak package of SiO_2_ aerogel disappeared in the XRD pattern of the nanoporous Ni obtained after removing the template. Meanwhile, within the measurement range (10°–90°), there were three strong diffraction peaks at 2θ = 44.58°, 51.92° and 76.50° which can be indexed to the characteristic (111), (200) and (220) crystalline planes of fcc Ni crystal, respectively. It also should be noticed that no other impurity phases, such as NiO*x* and Pd, can be observed, suggesting that the purity of nanoporous Ni is rather high.

Figure 6f shows the hydrogen uptake isotherms of the nanoporous Ni and Ni powder at room temperature. The amount of hydrogen increased with the increase of hydrogen pressure and the hydrogen storage capacity of nanoporous Ni was as high as 0.45 wt % at 4.5 MPa. However, the hydrogen storage capacity of the Ni powder was rather low, just about 0.04 wt % at 4.5 MPa, similar to reports of pristine nickel oxide powder [26]. There were very small changes in the amount of hydrogen within the Ni powder with the increase of pressure, which means there was weak hydrogen storage capacity for Ni powder. The enhancement of hydrogen uptake capacity of nanoporous Ni could be attributed to two factors. For one thing, compared with pristine Ni powder, the nanoporous Ni had a high specific surface area, which provided a large number of sorption sites for hydrogen molecule. For another, hierarchical pore structure could be more favorable for hydrogen adsorption. The macroporous component constructed from flower-like ultrathin nanosheets could be useful for fast transfer of hydrogen molecules during the adsorption-desorption process, while the microporous and mesoporous components formed by the mutual accumulation of nickel nanoparticles could accomplish sufficient adsorption of hydrogen [18]. Table 3 summarizes the hydrogen storage capacity of some hydrogen storage materials at room temperature in recent literature. It can be seen that the hydrogen storage capacity of the nanoporous Ni is comparable to some of the best hydrogen storage materials ever reported when compared to similar porous materials. It is particularly worth mentioning that pores with diameters of around 0.7 nm provided a high hydrogen adsorption per unit specific surface area [34]. And it was demonstrated in the experiments that the surface area directly affected the hydrogen adsorption capacity [35]. Additionally, from Table 3, it also can be seen that surface area plays a key role in hydrogen adsorption capacity. Thus, it can be concluded that the nanoporous Ni with high surface area and hierarchical pore structure is a desirable material for hydrogen storage. 

## 4. Conclusions

In summary, we have demonstrated a facile preparation method of nanoporous Ni by using SiO_2_ aerogel as template. The structure and magnetism of nanoporous Ni depended on the electroless plating time and temperature. The nanoporous Ni showed lower saturation magnetization (29.11 emu/g) and enhanced coercivity (321.78 Oe), which means nanoporous Ni prepared by this method is a desirable magnetic material in the application of data storage devices. The resultant nanoporous Ni obtained after electroless plating 6 times at 35 °C exhibits the high specific surface area of 120.5 m^2^/g and special porous flower-like structure consisting of Ni nanoparticles and ultrathin sheets. This nanoporous Ni can be used for hydrogen storage and exhibited a high hydrogen capacity of 0.45 wt % at 4.5 MPa at room temperature. 

## Figures and Tables

**Table 1:** Comparison of textural characteristics of the nanoporous Ni at different electroless plating conditions determined from nitrogen adsorption-desorption isotherms.

Samples	S_BET_ (m^2^/g)	Total Volume (cm^3^/g)	Average Pore Size (nm)
Ni-1	76.79	0.424	22.12
Ni-2	120.54	0.761	25.25
Ni-3	13.48	0.065	19.25
Ni-35 °C	120.54	0.761	25.25
Ni-50 °C	40.48	0.347	34.29

**Table 2:** Saturation magnetization (Ms), remnant magnetization (Mr), and coercivity (Hc) of the nanoporous Ni with different electroless plating times at 35 °C and bulk Ni.

Samples	Ms (emu/g)	Mr (emu/g)	Hc (Oe)	Resource
Ni-1	36.52	10.5	331.11	This work
Ni-2	29.11	7.8	321.78	This work
Ni-3	51.76	2.7	60.62	This work
Bulk Ni	55	2.7	100	[33]

**Table 3:** The hydrogen storage capacity of nanoporous Ni with different surface areas and the hydrogen storage capacity of other similar porous materials [18,35].

Samples	Surface Area (m^2^/g)	H_2_ Pressure (MPa)	Hydrogen Storage Capacity (at Room Temperature) (wt %)	Resource
Ni-1	76.79	4.5	0.27	This work
Ni-2	120.54	4.5	0.45	This work
Ni-3	13.48	4.5	0.08	This work
(Ni_0.347_Mn_0.346_Co_0.307_)O	--	3.1	0.42	[35]
(Ni_0.924_Co_0.021_Zn_0.055_)O	--	3.1	0.71	[35]
carbon aerogel	--	6.0	0.28	[18]
graphene aerogel	--	6.0	0.18	[18]

## Supplementary Materials

The following are available online at http://www.mdpi.com/2079-4991/8/6/394/s1.
