# Supplementary material for: Nanoporous Ni with High Surface Area for Potential Hydrogen Storage Application
Source: Nanomaterials (Basel). 2018 Jun 1;8(6):394. doi: 10.3390/nano8060394 (PMC6027325; doi:10.3390/nano8060394)
Supplement: Supplementary file 1 [file nanomaterials-08-00394-s001.pdf]

nanomaterials

MDPI

Supplementary Information

Nanoporous Ni with High Surface Area for Potential Hydrogen Storage Application

Xiaocao Zhou [1,2], Haibo Zhao [3], Zhibing Fu [2], Jing Qu [1], Minglong Zhong [2], Xi Yang [2], Yong Yi [1,*] and Chaoyang Wang [2,*]

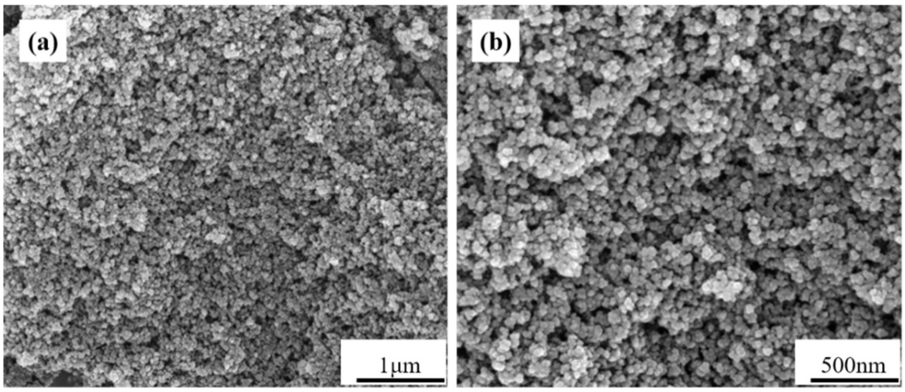

Figure S1. SEM images for SiO_2 aerogel.

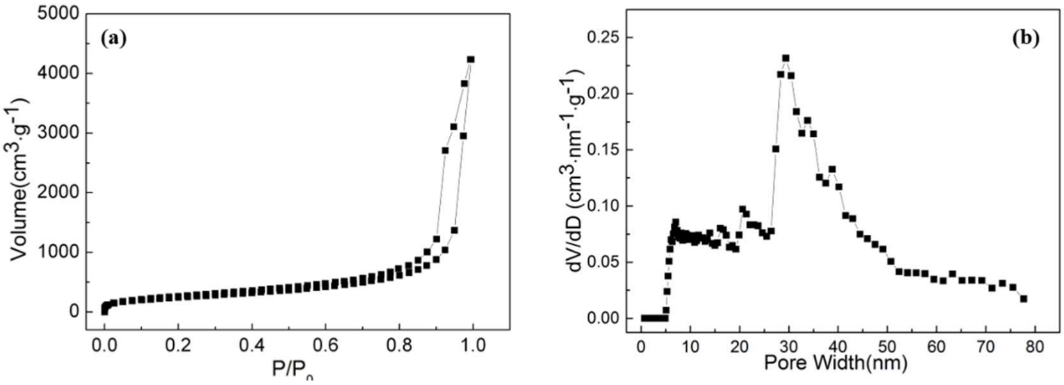

Figure S2. Nitrogen sorption isotherms (**a**) and size distribution (**b**) for SiO_2 aerogel.

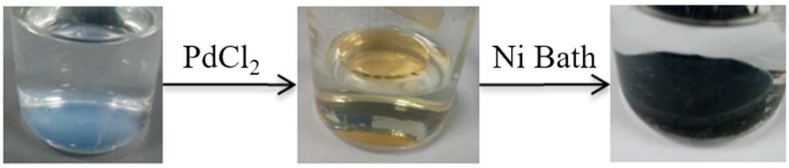

Figure S3. Chemical plating processes of Ni in the SiO_2 aerogel.

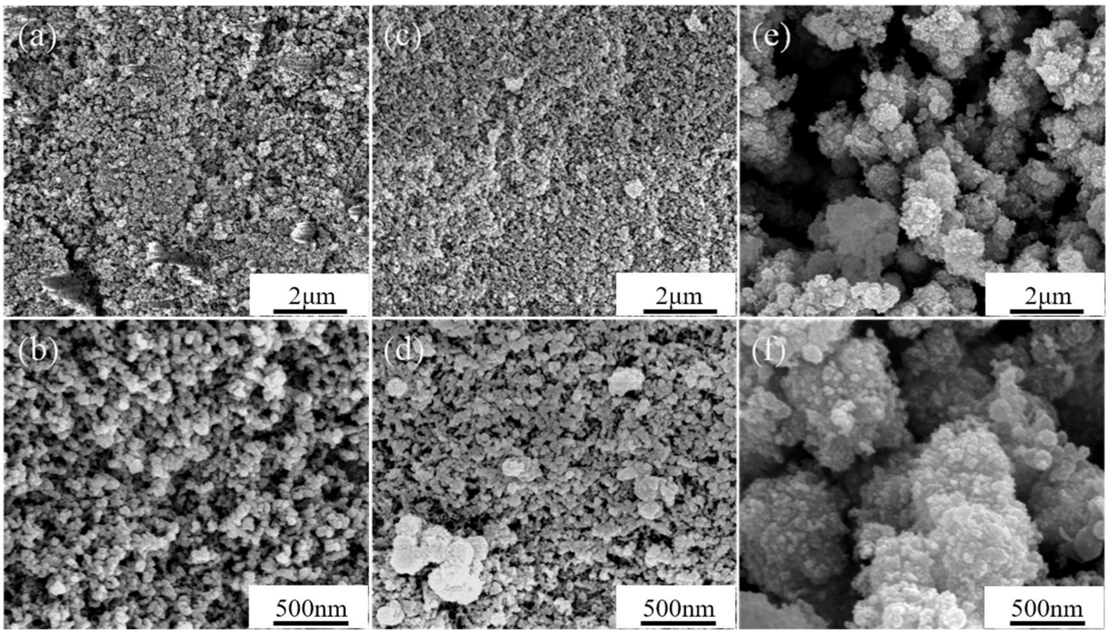

Figure S4. SEM images for SiO_2/Ni composites.

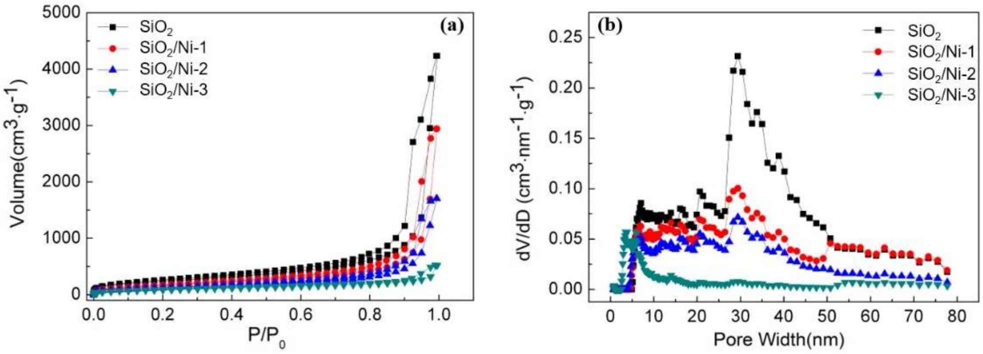

Figure S5. Nitrogen sorption isotherms (**a**) and size distribution (**b**) for SiO_2/Ni composites.

Table S1. Comparison of textural characteristics of the SiO_2/Ni composites at different electroless plating time at 35℃ determined from nitrogen adsorption-desorption isotherms.

Samples	S_{BET} (m²/g)	Total Volume (cm³/g)	Average pore size (nm)
SiO_2	890.64	6.59	29.50
SiO_2/Ni-1	606.01	4.56	30.11
SiO_2/Ni-2	455.71	2.64	23.20
SiO_2/Ni-3	325.37	0.81	9.96

Table S2. Ni content and O content for nanoporous Ni analysed by EDS, ICP-OES and XPS.

Samples	Ni content from EDS (wt %)	O content from EDS (wt %)	Ni content from ICP-OES (%)	Ni content from XPS (at %)
Ni-1	76.15	5.05	95.23	82.96
Ni-2	76.97	5.19	96.10	84.42
Ni-3	74.28	4.46	93.47	80.62

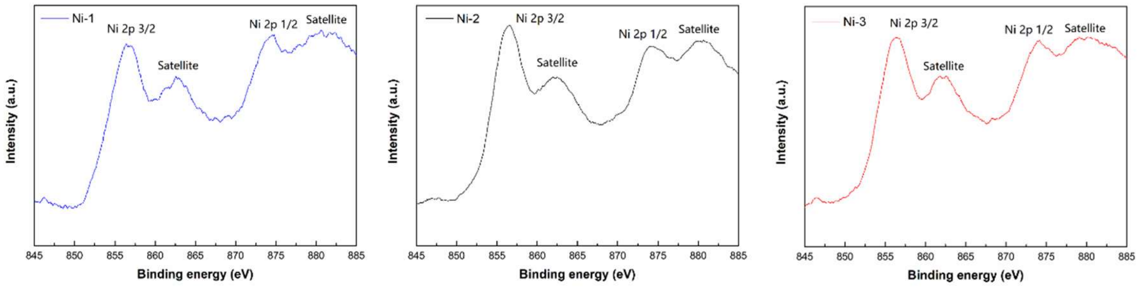

Figure S6. XPS spectrum of Ni 2p region for nanoporous Ni.

In order to investigate the surface composition of nanoporous Ni, XPS was used to determine the chemical state of the product. The Ni 2p spectrum are shown in Figure S6, in which two major peaks at 856.15 eV and 874.16 eV are indexed to Ni^{2+} of $Ni(OH)_2$ formed on the surface of nanoporous Ni during the removal of template under alkaline condition. Meanwhile, combined the results of EDS and ICP-OES, it can be seen that the amount of $Ni(OH)_2$ is very low, and there is almost no existence of NiOx in the spectrum of XRD. Therefore, we can conclude that nanoporous Ni is mainly composed of crystalline Ni.

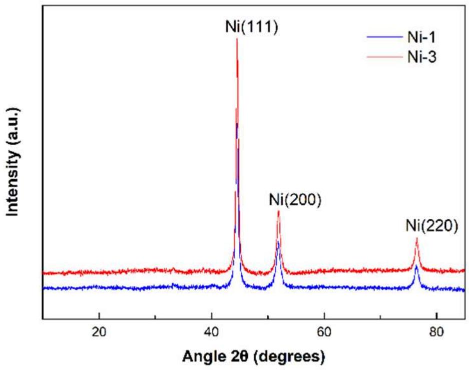

Figure S7. XRD spectrum for nanoporous Ni.

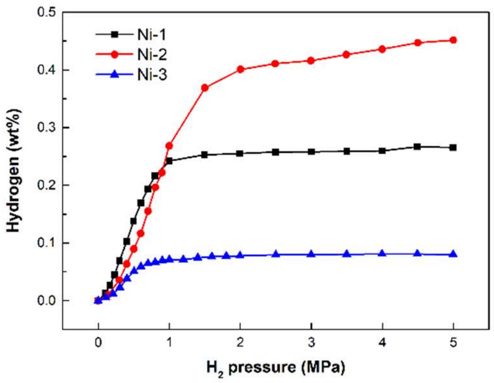

Figure S8. Hydrogen adsorption curves for nanoporous Ni.
